# Acute Effects of a Five-Minute Virtual Reality Nature Microbreak on Autonomic Function and Mood in Healthcare Workers

**DOI:** 10.7759/cureus.90341

**Published:** 2025-08-17

**Authors:** Yasumasa Oka, Takumi Jiroumaru, Kenji Mori, Shun Nomura, Hikaru Kitagawa, Takamitsu Fujikawa

**Affiliations:** 1 Rehabilitation, Kanazawa Orthopaedic and Sports Medicine Clinic, Ritto, JPN; 2 Physical Therapy, Bukkyo University, Kyoto, JPN

**Keywords:** healthcare workers, heart rate variability (hrv), microbreak, mood, virtual reality (vr)

## Abstract

Objective

The objective of this study is to investigate the acute effects of a five-minute virtual reality (VR) nature microbreak on autonomic function and mood in healthcare workers (HCWs).

Methods

Seventeen HCWs from an orthopedic outpatient clinic (mean ± SD age, 30.9 ± 7.9 years) completed a single-group crossover pilot study. Participants performed (i) a VR-guided paced breathing session displaying 360° natural scenery (VR condition) and (ii) an eyes-closed (EC) paced breathing session using an eye mask (EC condition) on separate days, at least 24 hours apart, during the same time slot (15:00-16:00). Breathing tempo was fixed at six breaths · min⁻¹ (four-second inhalation and six-second exhalation) using an auditory metronome. Five-minute photoplethysmographic heart rate variability (lnRMSSD, lnHF, lnTP, and lnLF/HF) and the short form of the Two-Dimensional Mood Scale (TDMS ST: Vitality, Stability, Pleasure, and Arousal) were recorded pre- and post-intervention. Post-pre differences were compared between conditions using paired t-tests. Effect sizes are expressed as Hedges’ g or Wilcoxon’s r.

Results

VR produced small, nonsignificant increases in autonomic indices (HF 0.17 ± 0.67 vs -0.05 ± 0.71 log ms²; t(16) = 1.35, p = 0.20, g = 0.31). Significant improvements were observed in the following TDMS subscales: Vitality (0.88 ± 3.22 vs -1.88 ± 3.98; p = 0.015, g = 0.63), Stability (4.53 ± 3.59 vs 2.12 ± 1.83; p = 0.025, g = 0.57), and Pleasure (5.41 ± 5.11 vs 0.24 ± 3.65; p = 0.005, g = 0.75). Arousal showed no significant difference (Z = -0.20, p = 0.84, r = 0.05).

Conclusions

A brief VR nature microbreak with paced breathing elicited medium-to-large improvements in subjective vitality, stability, and pleasure compared to conventional EC breathing, while producing only small, nonsignificant enhancements in parasympathetic HRV. VR microbreaks represent a feasible, low-cost, low-time intervention to boost immediate well-being in HCWs; however, larger and longer-term trials are warranted to confirm their physiological benefits and sustainability.

## Introduction

Healthcare workers (HCWs) perform their duties under considerable physical and psychological demands, making burnout a global concern [1,2]. Persistent occupational stress induces sympathetic dominance, resulting in reduced heart rate variability (HRV) and depressed mood, both of which impair clinical performance and increase patient safety risks [3,4].

One pragmatic countermeasure is to incorporate brief “microbreaks” of only a few minutes during work shifts to induce rapid relaxation [5]. However, studies evaluating the physiological and psychological effects of such breaks in clinical settings remain limited. Two practical metrics suitable for busy workplaces are HRV and the short form of the Two-Dimensional Mood Scale (TDMS ST). HRV quantifies autonomic activity noninvasively within minutes [6,7], whereas the TDMS ST assesses Vitality, Pleasure, Stability, and Arousal in under one minute, providing an instantaneous snapshot of affective state [8].

Recently, immersive virtual reality (VR) has gained attention as a microbreak modality. By presenting 360° natural scenes or meditation environments, VR can provide sensory isolation and may elicit deeper relaxation than conventional eyes-closed (EC) breathing [9]. Standalone head-mounted displays do not require external computers, making them easy to deploy in staff breakrooms. To our knowledge, no study has examined the simultaneous acute effects of VR-guided breathing on HRV and mood in HCWs during working hours.

We therefore prespecified our primary outcomes as changes in parasympathetic HRV indices (lnHF and lnRMSSD) and TDMS ST mood subscales. We hypothesized that, compared with EC paced breathing, a five-minute VR nature microbreak would produce small increases in parasympathetic HRV (Hedges’ g ≈ 0.3) and medium improvements in positive mood, particularly Vitality and Pleasure (Hedges’ g ≈ 0.5-0.8).

We selected a five-minute duration to align with microbreak literature (typically two to five minutes) and to balance physiological responsiveness with workplace feasibility [5]. Nature-based VR was chosen based on attention restoration and stress reduction frameworks, which predict greater affective restoration with natural stimuli than with non-natural or urban content [10].

## Materials and methods

Study design

This single-group crossover pilot study was conducted at an orthopedic outpatient clinic between May and June 2025. Each participant completed two microbreak conditions during a work shift between 15:00 and 16:00: (i) a VR condition, involving paced breathing while viewing 360° natural scenery through a headset, and (ii) an EC paced-breathing condition using an eye mask.

We used Nature Treks VR (Greener Games) on a Meta Quest 2 headset in seated mode with a stationary guardian boundary. Participants viewed the forest environment from a static, seated vantage point; all artificial locomotion (teleport/joystick movement) and camera translation/rotation were disabled, so the only scene motion arose from natural head tracking. To minimize optical flow and prevent simulator sickness, no scene movement or transitions occurred during the five-minute session, and particle/weather effects were set to the lowest in-app levels. The headset operated at the application’s default refresh rate (≥72 Hz) and default field-of-view; each participant adjusted the physical IPD slider as needed. Environmental audio consisted of low-intensity nature sounds delivered via in-ear earphones at a comfortable volume.

Participants sat upright on an armless chair with feet flat and hands resting on the thighs and were instructed to avoid speaking or voluntary movement during all recordings. Both conditions were conducted in a quiet meeting room (~23-25 °C).

During both sessions, participants followed a paced-breathing protocol. An auditory metronome track cued a cycle of 6 breaths·min⁻¹: a brief tone marked the start of inhalation (four seconds), followed by a silent countdown to the next cue (exhalation six seconds). The same audio file and volume were used in both conditions via earphones. The crossover sequence (VR→EC vs EC→VR) was generated using a computer-based random number list (permuted blocks of 4). Allocation was concealed in sequentially numbered, opaque, sealed envelopes, opened immediately before the first session by a coordinator not involved in data collection. The two sessions were scheduled on separate days with a washout period of ≥24 hours and were performed at the same time of day to control for circadian variability. Pre- and post-intervention assessments included five-minute HRV recordings and the paper-based Short Form TDMS ST. This was a pilot estimation study intended to quantify immediate effect sizes and feasibility; therefore, no a priori power analysis was performed for the initial sample of 17 participants. Based on the observed lnHF effect (Hedges’ g ≈ 0.31), a confirmatory trial would require approximately 80 participants to achieve 80% power.

Participants

Eligible participants were full-time HCWs (physical therapists, nurses, and medical clerks) aged 20-60 years employed at the clinic (≈30 staff; no night shifts). Exclusion criteria were (i) severe health conditions limiting normal duties (e.g., fractures, advanced musculoskeletal disorders, or malignancy); (ii) diagnosed cardiovascular or autonomic disease; (iii) regular use of β-blockers, antiarrhythmic agents, or benzodiazepines; (iv) a history of significant VR sickness or migraine triggered by VR; and (v) pregnancy or lactation. Recruitment was announced on the hospital bulletin board. Nineteen volunteers provided written informed consent; two were excluded due to incomplete HRV data, leaving 17 participants for analysis (13 physical therapists, four nurses; 10 men, seven women; mean ± SD age 30.9 ± 7.9 years).

Procedure and measures

HRV

HRV was measured using a finger-mounted photoplethysmograph (Pulse Analyzer Plus View; TAS9 View; YKC Corporation, Tokyo, Japan). Device validity has been previously reported [11]. Recordings were obtained after ≥2-hour postprandial rest; participants sat quietly for five minutes before sensor attachment and for an additional five minutes during each pre- and post-assessment. To reduce confounding factors, participants abstained from caffeine and nicotine for ≥3 hours, alcohol for ≥12 hours, and vigorous exercise for ≥2 hours prior to testing; regular medications were continued unchanged. HF power was used as the parasympathetic index, LF/HF as the sympathetic index, and total power (TP) as overall autonomic activity [12].

All HRV indices (RMSSD, HF, TP, and LF/HF) were computed using the device’s built-in artifact-detection/correction algorithm with default settings; values were then natural-log transformed (lnRMSSD, lnHF, lnTP, and lnLF/HF) in Excel (Microsoft Corporation, Redmond, WA, USA) for analysis.

Quality Control

We confirmed valid five-minute recordings without device error flags and screened for extreme outliers (>3 SD from the group mean). Two datasets were excluded because the device flagged irregular pulse waveforms or arrhythmic beats, precluding reliable five-minute HRV computation; no manual beat editing was performed. No additional segments were excluded.

TDMS ST

Concurrently with HRV measurement, participants completed the eight-item TDMS ST [8,13]. The instrument yields four subscales, Vitality, Pleasure, Stability, and Arousal, scored on a six-point Likert scale. The TDMS ST is not open-access; it was purchased from IMF Co., Ltd. (Tokyo, Japan), with permission for use granted by the developer (Y. Sakairi, personal communication).

Data analysis

Demographic variables (age, BMI, sleep hours/day, working hours/day, and years of clinical experience) were summarized as mean ± SD and checked for normality. Intervention effects were expressed as post-pre for each outcome. Normality was tested using the Shapiro-Wilk test. Paired t tests compared VR and EC conditions for normally distributed variables (RMSSD, HF, TP, LF/HF, Vitality, Pleasure, and Stability), while the Wilcoxon signed-rank test was applied to non-normal data (Arousal). Effect sizes were calculated using Hedges’ g (paired t) or r (Wilcoxon). Analyses were conducted in IBM SPSS Statistics for Windows, Version 28.0 (Released 2021; IBM Corp., Armonk, NY, USA); two-tailed p < 0.05 was considered significant. Analytic decisions (parametric vs. nonparametric) were prespecified based on normality rather than observed p-values; multiplicity adjustments were not applied, consistent with the pilot, estimation-focused design.

Ethical considerations

The study adhered to the Declaration of Helsinki and was approved by the Kanazawa Orthopedic Surgery Medical Clinic Ethics Committee (approval Kanazawa-OSMC-2024-007). All participants received written and verbal explanations of study aims, voluntariness, and data anonymization and provided written informed consent. Data were anonymized before analysis, and participants could withdraw at any time without penalty. Results are intended solely for academic purposes.

## Results

Seventeen participants completed the study and were included in the final analysis (13 physical therapists and four nurses; 10 men and seven women; mean ± SD age, 30.9 ± 7.9 years). Their baseline characteristics are summarized in Table 1.

**Table 1:** Baseline characteristics of the participants Sleep hours and work hours are expressed in hours per day.

Variable	Mean ± SD (n = 17)
Age	30.9 ± 7.9
Sex (men/women)	10/7
BMI	21.6 ± 3.0
Years of clinical experience	7.8 ± 4.6
Sleep hours	5.8 ± 1.3
Work hours	8.3 ± 0.8

HRV

Between-condition comparisons of post-pre values are presented in Table 2. All HRV indices increased in the VR condition compared with the EC condition, lnRMSSD (0.061 ± 0.309 vs -0.039 ± 0.498 log ms), lnHF (0.171 ± 0.668 vs -0.052 ± 0.710 log ms²), lnTP (0.149 ± 0.496 vs 0.070 ± 0.517 log ms²), and lnLF/HF (0.019 ± 0.208 vs -0.048 ± 0.205), but none reached statistical significance (largest difference: lnHF, t(16) = 1.35, p = 0.20). Effect sizes were small, with Hedges g = 0.31 for lnHF and g = 0.21 for lnRMSSD.

**Table 2:** Between-condition comparison of HRV (post-pre) EC, eyes-closed; HF, high-frequency power; HRV, heart-rate variability; LF/HF, low-frequency to high-frequency ratio; TP, total power; VR, virtual reality

Variable	VR (mean ± SD)	EC (mean ± SD)	t (16)	p-Value	Hedges’ g
RMSSD	0.061 ± 0.309	-0.039 ± 0.498	0.92	0.37	0.21
HF	0.171 ± 0.668	-0.052 ± 0.710	1.35	0.2	0.31
TP	0.149 ± 0.496	0.070 ± 0.517	0.63	0.54	0.15
LF/HF	0.019 ± 0.208	-0.048 ± 0.205	1.11	0.29	0.26

Mood (TDMS ST)

Condition comparisons for the TDMS ST are presented in Table 3. The VR microbreak produced significant improvements in three of the four subscales. Vitality: 0.88 ± 3.22 (VR) vs -1.88 ± 3.98 (EC); t(16) = 2.73, p = 0.015, g = 0.63. Stability: 4.53 ± 3.59 vs 2.12 ± 1.83; t(16) = 2.47, p = 0.025, g = 0.57. Pleasure: 5.41 ± 5.11 vs 0.24 ± 3.65; t(16) = 3.23, p = 0.005, g = 0.75. These correspond to medium-to-large effect sizes. Arousal showed no significant difference (Z = -0.20, p = 0.84, r = 0.05).

**Table 3:** Between-condition comparison of TDMS ST (post-pre) EC, eyes closed; TDMS ST, Two-Dimensional Mood Scale; VR, virtual reality

Variable	VR (mean ± SD)	EC (mean ± SD)	Test stat	p-Value	Effect size
Vitality	0.88 ± 3.22	-1.88 ± 3.98	t (16) = 2.73	0.015	g = 0.63
Stability	4.53 ± 3.59	2.12 ± 1.83	t (16) = 2.47	0.025	g = 0.57
Pleasure	5.41 ± 5.11	0.24 ± 3.65	t (16) = 3.23	0.005	g = 0.75
Arousal	-3.65 ± 4.51	-4.00 ± 5.01	z = -0.20	0.84	r = 0.05

## Discussion

This pilot crossover study is the first to evaluate the immediate effects of a five-minute VR-guided paced-breathing microbreak on both mood and autonomic function in clinical staff during work hours. Although VR produced only small, nonsignificant increases in HRV, it yielded medium-to-large improvements in the TDMS ST subscales: Vitality, Stability, and Pleasure. Minimal clinically important differences for TDMS ST have not been established in HCWs; therefore, these mood changes are interpreted primarily through standardized effect sizes and confidence intervals rather than clinical thresholds. This pattern, psychological indices responding faster than physiological ones, aligns with neuroimaging evidence that immersive VR can rapidly modulate emotion regulation networks, whereas autonomic adjustments emerge more gradually [14]. Because both mood and HRV were assessed at the same timepoints, our data do not establish temporal precedence between affective and autonomic responses; accordingly, the HRV findings should be regarded as inconclusive within the constraints of a five-minute microbreak. Prior research indicates that exposure to natural scenes activates parasympathetic pathways [10], consistent with the present gains of +0.88 points in Vitality and +5.41 points in Pleasure.

Several factors may explain why HRV changes did not reach statistical significance: (i) the small sample size of 17 limited statistical power; (ii) a five-minute intervention may be too brief to overcome large inter-individual variance in HRV; and (iii) breathing depth and thoracic movement were not monitored, although respiratory rate was fixed. With an SD of 0.67 for lnHF, approximately 80 participants would be required to detect the observed effect size (g = 0.31) with adequate power. Future studies should combine breathing-guidance applications with respiratory belts to provide real-time feedback and stabilize HRV responses.

No significant differences were observed in arousal. The VR content was deliberately configured with minimal optical flow to prevent simulator sickness, which may have limited arousal stimulation. Adjusting visual dynamics could allow content to be tailored for either relaxation or activation. Additionally, this trial was conducted in a day-shift outpatient clinic; HRV changes are reportedly larger during night shifts or in high-acuity wards [15,16], where VR microbreaks might exert stronger physiological effects.

From a practical standpoint, the intervention requires only a headset, lasts five minutes, and even if performed twice daily, demands less than 50 minutes per week. Compared with conventional mindfulness programs (20-30 minutes, two to three times weekly), VR microbreaks are time-efficient and require no specialist coaching, facilitating adoption. This comparison highlights time efficiency and implementability, without implying equivalent efficacy. Even small HRV improvements may accumulate with repeated use, suggesting that longitudinal trials over two to four weeks are warranted. Our cross-sectional design cannot assess the durability of autonomic changes, emphasizing the need for longitudinal confirmation.

Limitations include (i) a single-site, predominantly young Japanese cohort, limiting generalizability; (ii) variability in daily workload despite randomization; (iii) lack of long-term follow-up; and (iv) reliance on the device’s proprietary artifact-correction parameters (TAS9 View), limiting full reproducibility. Participants could not be blinded to VR exposure, so expectation bias cannot be excluded; this was partially mitigated by using identical paced-breathing prompts, earphones, and timing across conditions. EC paced breathing was chosen as a pragmatic, minimal-equipment comparator widely used in clinics; future trials should include active controls (e.g., non-nature VR or standardized relaxation audio) to isolate the contribution of immersive nature content. Primary endpoints captured immediate post-intervention effects; follow-up assessments (e.g., 10-15 minutes post and end of shift) will be incorporated to assess persistence. Multicenter studies with ≥100 participants, including night-shift and high-stress units, and ≥4-week repeated-use protocols are recommended. Future studies should also export beat-to-beat HRV series and process them using open, standardized software (e.g., Kubios HRV) to enable complete reporting of artifact thresholds and filters.

Future research should incorporate multimodal outcomes such as salivary cortisol and task performance. Exclusion of two device-flagged arrhythmic recordings may bias the sample toward more stable cardiovascular patterns; this exclusion was prespecified to preserve five-minute HRV validity and will be addressed by reporting beat-level quality metrics in future work.

In summary, a five-minute VR nature microbreak delivered during work shifts produced meaningful, immediate enhancements in positive mood and a small trend toward increased parasympathetic activity. Given the continuing reduction in VR costs, such microbreaks could become a practical and rapid stress management tool embedded in routine staff wellness programs.

## Conclusions

A five-minute VR nature microbreak with paced breathing produced immediate, medium-sized improvements in Vitality, Stability, and Pleasure, and a small, nonsignificant enhancement of parasympathetic HRV compared with EC control in HCWs. Because the intervention is brief, inexpensive, and requires only a standalone headset, VR microbreaks are highly deployable as an on-shift stress management tool. Larger, multicenter trials with extended follow-up are warranted to confirm physiological benefits and evaluate long-term outcomes. These findings should be viewed as exploratory estimates of immediate effects rather than evidence of clinical benefit, pending validation of clinically meaningful thresholds and longitudinal outcomes.
